# The inverted free energy landscape of an intrinsically disordered peptide by simulations and experiments

**DOI:** 10.1038/srep15449

**Published:** 2015-10-26

**Authors:** Daniele Granata, Fahimeh Baftizadeh, Johnny Habchi, Celine Galvagnion, Alfonso De Simone, Carlo Camilloni, Alessandro Laio, Michele Vendruscolo

**Affiliations:** 1 International School for Advanced Studies (SISSA), 34136 Trieste, Italy; 2 Institute of Computational and Molecular Science (ICMS), Temple University, Philadelphia, PA 19122, USA; 3 Department of Chemical Engineering, Massachusetts Institute of Technology (MIT), Cambridge, MA 02139, USA; 4 Department of Chemistry, University of Cambridge, Lensfield Road, Cambridge CB2 1EW, UK; 5 Division of Molecular Biosciences, Imperial College London, London SW7 2AZ, UK

## Abstract

The free energy landscape theory has been very successful in rationalizing the folding behaviour of globular proteins, as this representation provides intuitive information on the number of states involved in the folding process, their populations and pathways of interconversion. We extend here this formalism to the case of the Aβ40 peptide, a 40-residue intrinsically disordered protein fragment associated with Alzheimer’s disease. By using an advanced sampling technique that enables free energy calculations to reach convergence also in the case of highly disordered states of proteins, we provide a precise structural characterization of the free energy landscape of this peptide. We find that such landscape has inverted features with respect to those typical of folded proteins. While the global free energy minimum consists of highly disordered structures, higher free energy regions correspond to a large variety of transiently structured conformations with secondary structure elements arranged in several different manners, and are not separated from each other by sizeable free energy barriers. From this peculiar structure of the free energy landscape we predict that this peptide should become more structured and not only more compact, with increasing temperatures, and we show that this is the case through a series of biophysical measurements.

The free energy landscape of a protein provides a direct representation of the probability of measuring particular values of specific parameters that describe its conformational properties. The knowledge of the free energy landscape of a protein offers therefore the possibility of rationalising important aspects of its behaviour, including its stability, mechanisms of folding and molecular recognition, and the possibility of misfolding and aggregation^1^,^2^,^3^,^4^,^5^,^6^,^7^. It is thus highly desirable to extend this type of description to intrinsically disordered proteins, which are characterized by the absence of a well-defined structure under native conditions and play key roles in a variety of biochemical processes, including in particular signaling and regulation^8^,^9^,^10^,^11^,^12^,^13^. By bringing together experiments and computer simulations it has been recently possible to make great progress in characterizing the conformational space of this class of proteins^14^,^15^,^16^,^17^,^18^,^19^,^20^,^21^,^22^,^23^,^24^. Many questions, however, remain open about their behavior, including how they can access partially structured conformations to bind their partners and why they undergo progressive compaction upon increasing temperatures^25^,^26^,^27^,^28^.

In this work we show that molecular dynamics simulations within the framework of the free energy landscape formalism provides opportunities to answer these questions in the case of the monomeric form of the Aβ40 peptide, a 40-residue protein fragment associated with Alzheimer’s disease, which has been extensively studied experimentally and computationally^29^,^30^,^31^,^32^,^33^,^34^,^35^,^36^,^37^,^38^,^39^,^40^. Three main features of the Aβ peptide have emerged from these studies: (1) this peptide has an intrinsically disordered nature, (2) it tends to have higher aggregation propensity at increasing temperatures, and (3) its conformational properties are highly sensitivite to variations in the experimental conditions. For example, it has been reported that organic cosolvents tend to favour α-helical conformations and prevent aggregation^41^,^42^, and that high temperatures promote β-sheet formation and aggregation^43^.

To characterize the structure and thermodynamics of this peptide, we extend to intrinsically disordered proteins a recently introduced approach that enables the characterisation of the free energy landscape of folded proteins by using NMR measurements to accelerate the conformational sampling^44^ in explicit solvent all-atom simulations. In this strategy, NMR chemical shifts are used to define a collective variable that is then used in a bias-exchange metadynamics framework^45^. As in this approach the chemical shifts are not used as structural restraints, the resulting free energy landscape obeys the Boltzmann distribution corresponding to the force field used in the simulations. To investigate the idea that intrinsically disordered proteins become more ordered at high temperatures, in the simulations discussed here we considered a temperature of 350 K, where the is expected to be most evident. This choice has also the further advantage of speeding up the sampling of the conformational space. Then, to extend the results to more physiological temperatures, we also show how the population of the relevant states change with temperature through further calculations and experiments. Our results indicate that the global minimum in the free energy landscape of the Aβ40 peptide is represented by highly unstructured conformations, almost completely devoid of persistent secondary and tertiary elements. At higher free energies, however, partially structured conformations are present. What is particularly remarkable is the variety of topologies and secondary structure content of these states, which cover a wide range of different folds. In most of the cases these structures are not separated by significant free energy barriers, but belong to a single large basin, with the disordered structures at the bottom. As such partially folded conformations can be explored through thermal fluctuations at room temperatures, they can play the role of intermediates for molecular recognition or self-association. In this respect, these intermediates are reached from the global minimum through partial folding events, rather than through partial unfolding events, as is the case for folded proteins^46^,^47^,^48^.

A possible consequence of these results is that for increasing temperatures, when higher free energy states becomes more populated, intrinsically disordered proteins become more structured - and hence more compact - as their high free energy states are characterized by the presence of more secondary and tertiary interactions than their native states. We show that this scenario is indeed realized for the Aβ40 peptide by using a range of experimental biophysical techniques, which enable us to detect an overall structuring and compaction with increasing temperatures. We finally provide a thermodynamic analysis of the interplay between entropy and enthalpy that underlies this phenomenon.

## Results

We performed molecular dynamics simulations of the Aβ40 peptide in explicit solvent using NMR-guided metadynamics^44^. We thus obtained an estimate of the free energy as a function of a set of collective variables (see Methods). By using this approach one can estimate the free energy of all the relevant states explored during the dynamics, and compute the multidimensional free energy landscape of the system^44^. The errors in these free energies derive from those in the one-dimensional biases (displayed as error bars in Fig. S1), and are of the order of 2–3 kJ/mol, which is of the order of magnitude of thermal fluctuations, showing that the simulations reached convergence.

### The free energy landscape of the Aβ40 peptide

**Figure 1:**
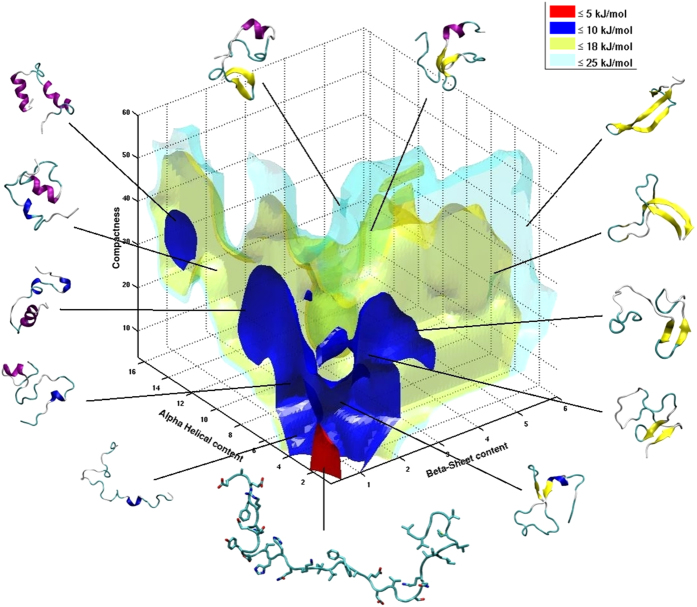
Free energy landscape of the Aβ40 peptide. The free energy landscape is shown as a function of three collective variables used in the NMR-guided metadynamics simulations: anti-parallel β-sheet content (X-axis), α-helical content (Y-axis) and number of hydrophobic contacts (or compactness, Z-axis). Isosurfaces are shown at 5 (red), 10 (blue), 18 (yellow) and 25 kJ/mol (cyan); white regions are not visited as they have higher free energies. Representative structures sampled during the simulation are also shown.

**Figure 2:**
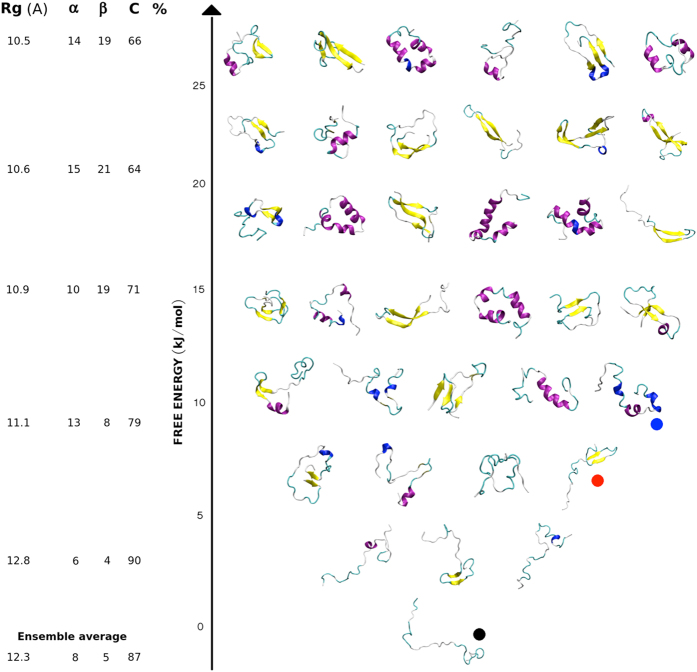
Characterization of the high free energy structures of the Aβ40 peptide. Representative conformations sampled during the simulations are shown at increasing values of free energy with respect to the global disordered minimum. On the left we report the average values of radius of gyration and secondary structure population for the whole ensemble and for difference slices in the free energy with a width of 6 kJ/mol (see also Figs 3 and 4). For increasing free energies, Aβ40 becomes more structured by populating both α-helical and β-sheet conformations.

From the molecular dynamics simulations described above we obtained a free energy landscape projected on three variables, the β-sheet content, the α-helical content and the number of hydrophobic contacts (Fig. 1). As expected from the intrinsically disordered nature of Aβ40 the global minimum in this free energy landscape corresponds to an ensemble of highly disordered structures, with only a few transient tertiary contacts and almost no secondary structure elements. Remarkably, however, we also found that at higher free energies the free energy landscape includes a wide variety of partially structured conformations. The free energy of these partially folded conformations is in many cases only 8–10 kJ/mol higher than that of the global minimum, indicating that their populations are not negligible. Moreover, these structures are characterized by several different topologies (Figs 1 and 2) including α-helixes and β-sheets in several different combinations. These structures do not correspond to significant local free energy minima, as shown in Fig. 1. Indeed, the free energy landscape resembles a wide basin, without any sizable free energy barrier, where all the states are kinetically committed to the disordered free energy minimum and can exchange rather rapidly among each other.

To obtain a more quantitative description of the free energy landscape we calculated, for increasing values of the free energy, the radius of gyration and secondary structure populations (see Methods). Our results indicate that the overall shape of the free energy landscape of Aβ40 appears qualitatively inverted with respect to that of globular proteins. The structural ensemble that characterizes the global minimum in the bottom region of the free energy landscape has relatively high values of the radius of gyration (13 Å) and no sizeable secondary structure element (90% random coil population), rather than a well-defined structure. The amount of secondary structure gradually increases with the free energy, with a corresponding decrease in the radius of gyration, in a manner opposite to what happens in folded proteins, for which the native state structure is gradually lost in the states at higher free energies.

### Secondary structure characterization

**Figure 3:**
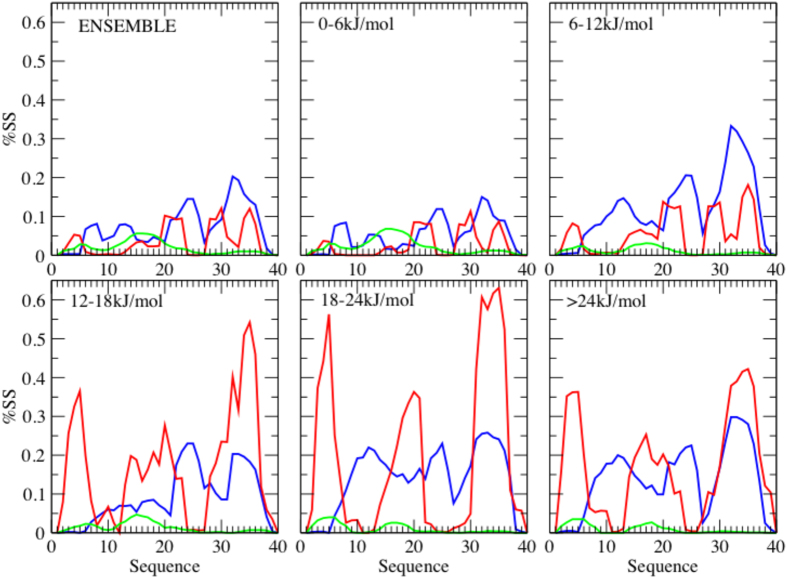
Secondary structure populations of the Aβ40 peptide at increasing values of the free energy. The lines indicate different secondary structure types: α-helical regions are shown in blue, β-sheet regions in red, and polyproline II regions in green. The different panels report the secondary structure populations (‘%SS’, which is given as a fraction of the total population, i.e.from 0 to 1) corresponding to different slices of the free energy landscape: (**a**) entire free energy landscape, (**b**) lower region of the free energy landscape (0–6 kJ/mol), (**c**) 6–12 kJ/mol region, (**d**) 12–18 kJ/mol region, (**e**) 18–24 kJ/mol region and (**f**) higher region (above 24 kJ/mol).

The picture described above is supported by a detailed analysis of the secondary structure propensity for each residue (Fig. 3). An ensemble average (Fig. 3a) shows an overall low secondary structure content, with α-helical conformations favoured with respect to β-strand ones, consistent with previous results^33^,^37^. However, as the free energy progressively increases (Fig. 3b–f), the observed secondary structure populations increase accordingly, following a characteristic sequence-dependent pattern. The residues that are more prone to form α-helices are found primarily in the second half of the protein (residues 21–26 and 30–37), and also to a smaller extent in the first half (residues 7–15). While the α-helical content remains rather stable above the bottom region of the free energy (Figs 2, and 3c–f), the β-strands population starts to grow at increasing free energies and is concentrated in three different regions, at the two termini and in the middle of the sequence, as also found previously^37^. Also some polyproline II conformations, typical of intrinsically disordered proteins^26^, are sampled between residues 11 and 20 in the most disordered and lowest free energy region of the landscape (Fig. 3b), and then disappears in the higher free energy windows.

This characterization of the free energy landscape is consistent with the heterogeneous structural behaviour described in previous studies for different experimental conditions. At 5 °C and pH 7.1 residual β-strands populations were observed in the central and C-terminal region (17–21 and 31–36) and no sizeable α-helical population^49^. By contrast, in the presence of an organic cosolvent mimicking membrane-like conditions and at low pH, α-helical conformations appeared to be favoured^49^. At 25 °C and pH 2.8 in a TFE/water mixture the presence of two distinct α-helical regions was detected for residues 15–22 and 31–35^41^. Under similar conditions (at 25 °C and pH 5 in water-micelles environment), an extended α-helical conformations was described in the C-terminal part of the peptide (residues 15–36), interrupted by a hinge region between residues 25 and 27^42^, which exhibits a similar behavior in the parallel β–sheet arrangement of the central and C-terminal regions of the amyloid fibrils formed by this peptide^50^. The smooth and barrierless shape of the free energy landscape, with many partially folded states at similar low free energies, provides an explanation to the high sensitivity of the system to the environmental conditions. Indeed, above the global disordered minimum, specific perturbations can readily shift the populations favouring a variety of different states.

### Solvent exposed surface area

**Figure 4:**
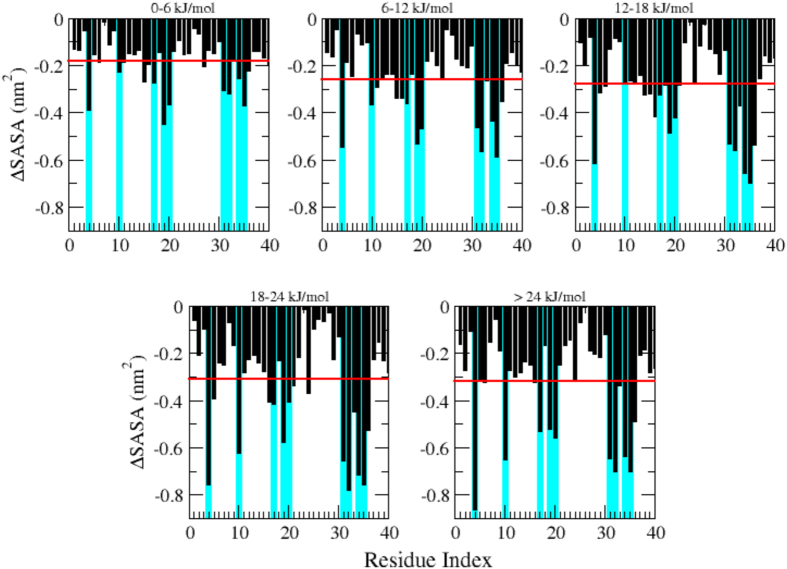
Residue-based analysis of solvent accessible surface area (SASA) of Aβ40 at increasing values of the free energy. Black bars correspond to the SASA difference with the conformations in the global minimum for each residue averaged on the same free energy windows. The horizontal red line shows the average SASA value for the sequence, which is progressively increasing with the free energy. This effect is particularly evident for all the larger hydrophobic residues, highlighted by cyan bars in background.

For each conformation in the ensemble, we also computed the difference of the solvent accessible surface area (SASA) for each residue with respect to the configurations corresponding to the global free energy minimum. We observed a systematic decrease in the exposure for all the residues as the free energy increases (Fig. 4). This effect is particularly strong for the larger hydrophobic residues in the sequence (Phe4, Tyr10, Leu17, Phe19, Phe20, Ile31, Ile32, Leu34, Met35). Remarkably, these residues belong to regions where the probability of observing β-sheets is high (Fig. 3). These results suggest that β-strands can promote the protection of hydrophobic side chains from solvent exposure. One could expect this effect to be less readily achievable through α-helical conformations, as it would involve the formation of stable tertiary contacts such as those formed in globular proteins. In membrane-like environments, where the organic cosolvents are able to screen the hydrophobic residues, β-sheets conformations have not been observed and these residues appear to have a higher propensity to form α-helical conformations.

### Comparison of the ensemble of structures generated by molecular dynamics simulations with NMR measurements

We compared the structural properties of the ensemble of structures generated by molecular dynamics simulations with available NMR chemical shift measurements^49^. Since we have not used these measurements as structural restraints in the simulations, the free energy projection on the Camshift CV (Fig. S1A) indicates that the generated ensemble is broadly consistent with the experimental chemical shifts, as this CV measures the deviation from the experimental values. To investigate this aspect more quantitatively, we computed the average chemical shift of the backbone atoms using SPARTA+^51^ rather than Camshift to avoid possible systematic errors. The comparison between calculated and experimental chemical shifts indicates that, despite the differences in temperature between experiments and simulations, for all the species the values differ always by less than the typical accuracy of SPARTA+ (Table S1).

### Experimental measurement of the progressive structuring of Aβ40 upon increasing temperatures

**Figure 5:**
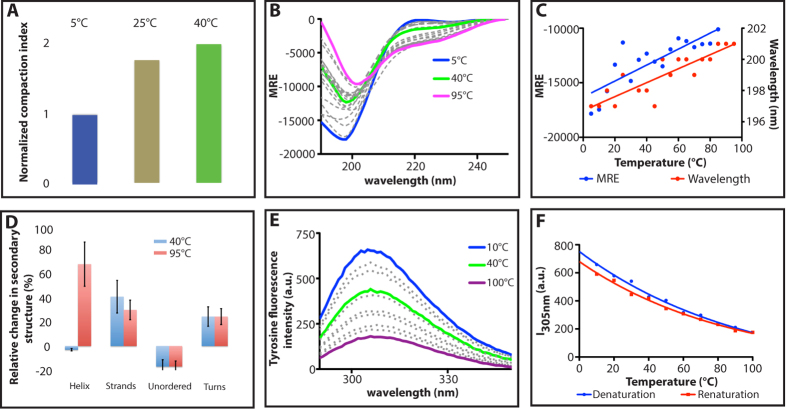
Temperature-induced compaction of the Aβ40 peptide in 20 mM phosphate buffer at pH 8. (**A**) Values of the compaction index (CI) relative to that at 5 °C, as calculated from the hydrodynamic radii determined by NMR diffusion experiments at a concentration of 200 μM. (**B**) Far UV-CD spectra recorded at a concentration of 20 μM. (**C**) CD spectra-based values showing the increase (MRE) and the red-shift (wavelength) of the minimum mean residue ellipticities with increasing temperature. (**D**) CD-derived secondary structure population showing the relative change of the α-helical, β-strand, unfolded and turn populations of Aβ40 between relative to those at 5 °C. (**E**) Quenching of tyrosine fluorescence intensity with increasing temperature. (**F**) Reversibility of the quenching of tyrosine fluorescence intensity followed at 305 nm between 5 and 100 °C.

Given the architecture of the free energy landscape described above, these computational results suggest the possibility of a remarkable phenomenon - at increasing temperatures the Aβ40 peptide should become more structured, as it could explore regions of higher free energy. To test experimentally this prediction, we used a combination of hydrodynamic and spectroscopic approaches. As anticipated from the metadynamics simulations described above, we show that the structuring of the Aβ40 peptide increases with temperature, supporting the notion that this peptide undergoes a temperature-induced partial folding (Fig. 5). The details of the experimental procedures are presented in the Methods section.

#### Size exclusion chromatography

Since the elution profile of a protein from a gel filtration column depends on its hydrodynamic properties, we used size-exclusion chromatography (SEC) to infer the hydrodynamic properties of the Aβ40 peptide by comparing its elution volume from the gel filtration column with those of known proteins^52^. The peptide was eluted at 20 °C from the SEC column as one sharp and symmetrical peak with an apparent molecular mass (MMapp) of 8.8 kDa, a value that is well above the theoretical one for a folded polypeptide chain of similar length (i.e. 4.4 kDa). According to ref. 53, the *R*_*h*_ of the Aβ40 peptide can be deduced from the MMapp and it is about 16 Å (Table 1). By comparing the *R*_*h*_ value of the Aβ40 peptide with the theoretical hydrodynamic radii expected for various conformational states of a polypeptide chain with a similar length as the Aβ40 peptide (F: natively folded, U: urea unfolded and PMG: pre-molten globule), the *R*_*h*_ value of the Aβ40 peptide was found to be similar to the expected value of a PMG-like conformation (whose expected value is 17 Å) (Table 1). Accordingly, the large MMapp and *R*_*h*_ values of the Aβ40 peptide are not compatible with a folded and monomeric globular structure. Rather, such large values can be attributed to an extended conformation with the low compactness typical of intrinsically disordered proteins^53^.

#### NMR diffusion

To directly assess the temperature effects on the conformational properties of the Aβ40 peptide, we used 1D pulsed-field gradient NMR diffusion experiments^54^. The very small spread of the resonance frequencies for amide protons (between 7.8 ppm and 8.7 ppm, data not shown) are typical of proteins without any stable secondary structure, thereby supporting the lack of a packed structural core within the Aβ40 peptide. The NMR-deduced *R*_*h*_ value of Aβ40 was found to be 15.9 Å at 25 °C (Table 1). In agreement with SEC analyses, this *R*_*h*_ value falls between the values corresponding to completely unfolded and natively folded states, thus further supporting the occurrence of residual transient secondary structures typical of intrinsically disordered proteins in the pre-molten globule state.

Interestingly, and in contrast to the typical behavior of folded proteins, increasing the temperature led to a gradual decrease of the *R*_*h*_ value, thus leading to a heat-induced conformational compaction. The *R*_*h*_ value decreased by about 8% between 5 °C and 40 °C (Table 1). At all measured temperatures, the *R*_*h*_ values correspond to pre-molten globule-like conformations with different levels of compaction. To quantitatively estimate the degree of compaction of the Aβ40 peptide, we used the compaction index (CI)^55^. The CI value can vary between 0 and 1, with 0 indicating essentially no compaction and 1 maximal compaction. These values were calculated using the reference *R*_*h*_ values for unfolded and natively folded proteins (see Methods). The CI values increased from 0.21 to 0.45 when the temperature was increased from 5 to 40 °C (Table 1 and Fig. 5A). These results further support the temperature-induced structuring of the Aβ40 peptide.

#### Circular dichroism

The far-UV CD spectra of the Aβ40 peptide are typical of unstructured proteins, as seen from their large negative ellipticities at 200 nm and very low ellipticities at 190 nm (Fig. 5B). However, the observed ellipticities at 200 and 222 nm indicate that they are not fully unfolded and rather conserve some residual transient secondary structures, in agreement with hydrodynamic analyses. In order to monitor heat-induced structural changes that may occur within the Aβ40 peptide and could possibly be responsible for the peptide chain structuring, we recorded CD spectra in a wider temperature range (5–95 °C) as the ones used for the NMR measurements (Fig. 5B), i.e. we considered a range that includes the value adopted in the simulation. The CD spectra of the Aβ40 peptide showed significant changes upon heating. Indeed, a considerable gain of negative ellipticity around 222 nm and a loss of negative ellitpicity around 200 nm were observed. Moreover, the minimum negative ellipticity did not only experience an increase in the intensity but underwent a slight but significant shift of about 5 nm towards higher wavelengths, in agreement with more structured conformations (Fig. 5C). These spectral changes reflect temperature-induced gain in secondary structures while the protein remains disordered. To estimate quantitatively the temperature-induced secondary structure formation, all spectra were subsequently deconvoluted using the CDSSTR method that is implemented in the CDPro software package for analyzing protein CD spectra (Fig. 5D)^56^. These analyses indicated a temperature-dependent increase of about 50% of β-strands and 27% of turns and a decrease of 16% of the unfolded fractions between 5 and 40 °C. On the other hand, in the same range of temperature, the α-helix content remained almost unchanged. Strikingly, however, when increasing the temperature to 95 °C, the α-helix content increases dramatically by 100% (i.e. it doubles), whereas the β-strand content decreases by 15% with respect to the values at 40 °C. The populations of turns and coils remained instead unaffected (Fig. 5D). These data are consistent with our prediction, as well as with the structural analysis of the free energy landscape in Figs 2 and 3.

#### Tyrosine intrinsic fluorescence

Intrinsic tyrosine fluorescence of the Aβ40 peptide showed an emission λ_max_ at 305 nm with an excitation peak at 275 nm. The scans upon increasing temperatures show a decrease in tyrosine fluorescence intensity (Fig. 5E). Indeed, tyrosine emission intensity at 305 nm (I305 nm) decreased by 35% when heating up to 40 °C and by 75% at 100 °C. As it has been established that the emission λ_max_ of tyrosine is independent from temperature^57^, the observed temperature-dependent decrease in the tyrosine intensity at 305 nm of Aβ40 monomers is attributable to effective tyrosine quencher interactions that are probably due to interactions between the excited chromophore and its environment as a result of the environmental changes around Tyr10, confirming what shown in the SASA analysis in Fig. 4. In further support, the fluorescence spectra show that the transitions involved are completely reversible, as the emission intensities at various temperatures were restored on renaturation (Fig. 5F).

Taken together these data suggest that increasing temperatures induce a progressive structuring of the Aβ40 peptide rather than the unfolding typical of globular proteins. The overall temperature-induced compaction, reported recently for intrinsically disordered proteins and the unfolded states of globular proteins^25^,^26^,^27^,^28^, is therefore determined here not by the formation of a basically disordered molten globule, but rather by the increase in population of more structured, and hence more compact, conformations. These results also offer a possible explanation for the observation of the high propensity of Aβ40 to form amyloid fibrils at physiological temperatures and a rather low propensity at lower temperatures^58^, due to the increasing of β-sheet population.

### Thermodynamic considerations on the temperature-induced structuring of the Aβ40 peptide

To investigate the mechanism underlying the progressive structuring of the Aβ40 peptide with temperature reported in this work, we performed three additional molecular dynamics simulations of 100 ns starting from three different conformations belonging to the lowest part of the free energy landscape: (C1) the unstructured global minimum (Fig. 2, black circle); (C2) a conformation with high β-sheet content with a free energy 8 kJ/mol higher than the minimum (Fig. 2, red circle); (C3) a conformation with high α-helical content at a free energy of 10 kJ/mol (Fig. 2, blue circle). Averaging the total force field energy over the three runs (see Methods for details), we verified that the enthalpy is significantly lower for the two structured states, by −60 ± 8 for C2 and by −28 ± 5 kJ/mol for C3 with respect to C1.

**Figure 6:**
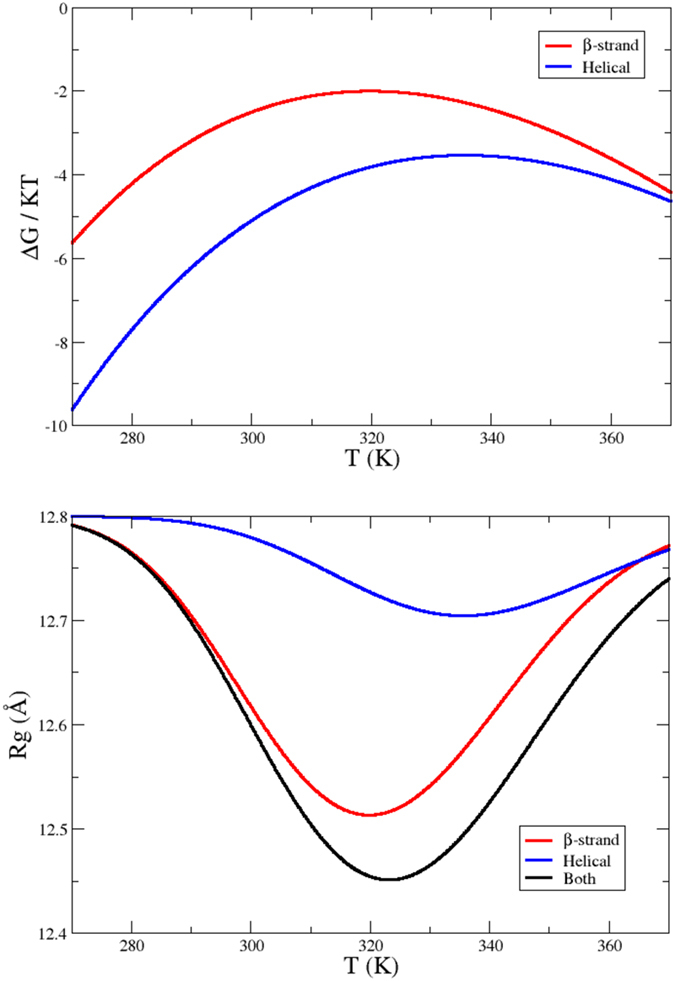
(**A**) Temperature-dependence of the free energy difference with the disordered global minimum for two structured conformations (respectively blue and red dots in Fig. 2) according the corresponding two-state model. (**B**) Temperature-dependence of the radii of gyration of these states.

We then considered the temperature dependence of the logarithm of the population ratio between the unstructured state C1 and, respectively, C2 and C3, obtained by applying a simple two-state model (see Methods for details) based on the enthalpy estimates (Fig. 6A). We found that the unstructured state C1 is favoured (ΔG < 0) in both cases, reflecting the intrinsically disordered nature of the peptide and the dominant role of entropy (see also Fig. S3). However the structured states increase their populations with temperature, reaching a maximum between 320 K and 340 K, consistently with the range of temperature-induced collapse observed for the unfolded states of other proteins^25^,^26^,^27^,^28^. These findings indicate that, for these intermediate temperatures, the free energy difference between the disordered global minimum and the structured configurations decreases, consistently with the structuring observed in the experiments. The low curvature of the two curves shows however that the free energy does not change abruptly with temperature, which is consistent with the modest decreasing of the hydrodynamic radius shown by experiments. The same trend is observed for the calculated radius of gyration restricted to a mixture of the three states C1, C2 and C3 (see Fig. 6B). We note also that C2 is dominant with respect to C1, as found in the experiments reported above about β-sheet and α-helical structures, as well as in other studies^49^. This scenario, together with the observation that hydrophobic residues show a higher propensity to form β-sheet conformations (Fig. 4), suggests that the structuring could be induced in this system by entropic contributions from the solvent, which become increasingly important at higher temperature. At intermediate temperatures the relative compaction produces a favourable solvation contribution to the free energy, which, together with the formation of hydrogen bonds in secondary structure elements, counterbalances the loss of conformational entropy of the peptide. At even higher temperature the latter becomes again dominant.

## Discussion

In this work we have presented a quantitative analysis of the free energy landscape of the intrinsically disordered Aβ40 peptide, obtained by converged molecular dynamics simulations in explicit solvent. The convergence in these calculations, which is in general extremely challenging in the case of intrinsically disordered proteins, was possible here by the use of a recently introduced enhanced sampling technique, NMR-guided metadynamics^44^. This approach provides an atomic-level characterization of the most relevant conformational states of the system, probing structural features that would not be readily accessible to standard experimental techniques, and is therefore particularly promising in the application to intrinsically disordered proteins. The results that we have reported are fully consistent with the intrinsically disordered nature of the Aβ40 peptide and reconcile the previous heterogeneous evidences about its secondary structure propensity. The availability of the relative free energy for a large number of conformations enabled us also to define a library of structures (Fig. 2) that may be used as targets to design inhibitors capable of binding Aβ40 peptides in order to prevent their aggregation^59^.

The free energy landscape that we observed, with a largely disordered global minimum and a variety of partially folded conformations only 8–10 kJ/mol higher in free energy, suggest a way to rationalize many of the characteristic properties of also other intrinsically disordered proteins. This type of architecture can provide to these proteins the ability of rapidly interconverting among different states, allowing them to perform signalling and regulatory tasks. Since in this description very different structures have similar free energies, specific environmental conditions or the presence of binding partners could readily modify the free energy landscape. These results may provide also an explanation for the observation of the progressive compaction of intrinsically disordered proteins with increasing temperatures^25^,^26^,^27^,^28^. The Aβ40 peptide undergoes a temperature-induced structuring since larger thermal fluctuations favour the population of higher free energy regions with higher secondary structure content (Fig. 3), as we have first predicted computationally (Fig. 2) and then validated experimentally (Fig. 5). Our calculations and experiments show that for the Aβ40 peptide this behaviour determines an increase in β-sheet propensity with temperature, which is associated with the burying hydrophobic residues (Fig. 4). This inverted role of temperature in driving a disorder-to-order transition for intrinsically disordered proteins with respect to folded proteins is consistent with the expectation that the landscape of the latter may be enthalpy-dominated, while the landscape of the former is instead entropy-dominated.

Despite these topological differences in the free energy landscapes of ordered and disordered proteins, the underlying thermodynamic behaviour would appear to be siimilar, although further studies will be necessary to confirm this possiblity. The curves in Fig. 6 can be considered analogous to the usual stability curves for folded proteins: even if the unstructured state is always favoured, we do observe a maximum in the population of structured states, due to their lower enthalpy, preceded and followed by an increasing predominance of the unstructured states. This phenomenon appears reminescent of the cold and heat denaturation in globular proteins^60^.

Although a larger number of cases have to be investigated to establish more general conclusions, the results that we have presented provide a glimpse of how the free energy landscape formalism can be adapted to describe also the case of intrinsically disordered proteins. The absence of a well-structured native state implies the presence of a variety of partially structured states. This is not the case for globular proteins, for which the presence of a structurally-defined native states appears to limit rather significantly the number of alternative partially structured states, that could act as kinetic traps in potentially aggregation-prone partially folded states. In this scenario the conformational fluctuations in unfolded states of globular proteins are preferentially directed towards the population of partially structured intermediates with native-like topologies, while the conformational fluctuations in intrinsically disordered states populate a wide range of different partially structured conformations. However, in both cases raising the temperature increases the population of metastable intermediates, which can provide access to aggregation pathways. For ordered proteins, such intermediates are explored through conformational fluctuations from the folded state^46^,^47^,^48^, while for disordered proteins they are accessed by partial ordering events, also caused by conformational fluctuations, from the broad ensemble of structures making up the global minimum in their free energy landscape.

## Methods

### NMR-guided metadynamics simulations

#### Simulation details and metadynamics parameters

Molecular dynamics simulations were performed in explicit solvent at 350 K using the CHARMM22* force field^61^ and the TIP3P water model^62^, employing the GROMACS 4.5.3 package^63^. The protein was solvated by 6461 water molecules and 3 sodium ions in a 203 nm^3^ dodecahedron periodic box. Long-range interactions were accounted for using the particle-mesh Ewald method^64^, with a short-range cutoff of 0.9 nm. All bond lengths were constrained to their equilibrium length with the LINCS algorithm^65^. The time step for the molecular dynamics simulation was set at 2.0 fs and the Nose-Hoover thermostat^66^,^67^ with a relaxation time of 1 ps was used. The simulations were run for 310 ns on eight replicas (for a cumulative simulation time of 2.5 μs). Each replica was biased on a single collective variable (CV) using a bias exchange scheme, where exchanges between the different replicas are periodically attempted according to a replica exchange scheme, and this process is repeated until convergence of the free energy profiles is obtained. The collective variables that we used quantify the α-helical, anti-parallel and parallel β-sheet contents^68^, the coordination number for hydrophobic side-chains, and the deviation from the average conformation of the residues side chains. Finally, we used two CamShift collective variables, defined using experimental chemical shifts (BMRB bmr17795). The functional form of these collective variables are as described previously in ref. 44. One-dimensional Gaussian functions of height w = 0.30 kJ/mol were added every 5 ps, and exchanges of the bias potentials were attempted every 20 ps. After 100 ns of simulation, in which very wide regions of the CVs were explored, we introduced loose upper boundaries to help the convergence of the bias potentials^69^. The metadynamics parameters for the different CVs have been previously benchmarked on several unbiased simulations starting from different conformations^44^. They are:

- Camshift Parameters: Gaussian width σ = 1.
- AlphaRMSD. Parameters: m = 8, n = 4, R_0_ = 0.1, σ = 0.2
- ParaBetaRMSD Parameters: m = 12, n = 6, R_0_ = 0.1, σ = 0.1
- AntiBetaRMSD. Parameters: m = 12, n = 6, R_0_ = 0.1, σ = 0.2.
- Coordination Number. Parameters: m = 8, n = 4, R_0_ = 0.4, σ = 10
- Two AlphaBeta Similarities. Parameters: σ = 0.3 for hydrophobic residues and σ = 0.075 for polar residues. All the CVs are implemented in the PLUMED plugin^70^ for GROMACS.

#### Free energy reconstruction in the CV space

Bias-exchange metadynamics allows the free energy of a system to be reconstructed once the bias potentials become stable. This happens in our case after an equilibration time *t*_*eq*_ = 150 ns. After selecting the CVs that are most effective to discriminate different states of the system, the CVs space is divided in hypercubes and all the simulation frames are assigned to the corresponding microstates according to its CVs value. The structures within each hypercube must be consistent to define a proper microstate of the system, otherwise its size must be reduced, doing again the assignment. Then a free energy value is computed for the microstate, according to the corresponding bias potentials and the populations observed after the *t*_*eq*_. In our study we have chosen the Coordination Number, AlphaRMSD, Anti- and Para-BetaRMSD CVs; the relative free energy profiles are reported in Fig. S1. From these calculations we estimate that up to 40 kJ/mol the free energies that we obtained are precise within 2–3 kJ/mol, comparable with thermal fluctuaction (kT = 2.9 kJ/mol). All the analysis has been performed as previously described using METAGUI, a VMD interface for analyzing metadynamics and molecular dynamics simulations. For a further description of the computational method see also ref. 44.

#### Ensemble averages

The description of a system in terms of its free energy landscape obtained by the bias-exchange metadynamics approach enables the calculation of any property (or observable *O*) of the system performing an ensemble average, having defined the microstates of the system and the corresponding free energies


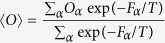


where the sums run over all the microstates, T is the simulation temperature and *O*_*α*_ is the average value of *O* on the structure which populate the microstates *α*. In this work we computed in particular the ensemble average of the backbone chemical shifts (Table S1), the secondary structure population and the solvent accessible surface area (SASA) per residue (see Figs 3 and 4).

#### Estimation of the enthalpy and of the temperature-dependence of the free energy

We performed three molecular dynamics simulations of 100 ns with the same setup described above starting from three different conformations from the reconstructed free energy landscape: (C1) the unstructured global minimum; (C2) a β-strand conformation with a free energy 8 kJ/mol higher than the minimum; (C3) a α-helical conformation at a free energy of 10 kJ/mol (see Figs 2 and 6). To evaluate the enthalpy of their corresponding thermodynamic states, we applied a harmonic restraint around the corresponding centroid in the four dimensional space of the CVs used for the free energy reconstruction. In this way the system remains around the original microstate, allowing an evaluation of its enthalphy (averaging the total force-field energy) and, thus, of the relative entropy, by difference with the known free energy of the state (see Fig. S2 for the convergence of the enthalpy estimate). We discarded from the analysis all the frames (acquired every 5 ps) where the restraint potential exceed the thermal fluctuations (2.9 kJ/mol). The restraint potential has been taken in account in the averaging of the total force-field energy.

The availabilty of the free energy, entalphy and entropy differences between the disordered global minimum and the two structured states enables the calculation of the corresponding free energy difference varies as a function of the temperature, applying a simple two-state model^71^. Instead of considering the unfolding free energy *ΔG*_*UNF*_ = *G*_*U*_ − *G*_*F*_, between the unfolded (*G*_*U*_) and the folded state (*G*_*F*_), we can use the difference between an unstructered (*G*_*U*_) and a structured state (*G*_*S*_). By assuming a constant heat capacity *C*_*p*_ 60, we have


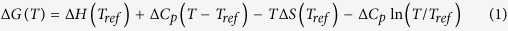


where the enthalpy and entropy differences *ΔH(T*_*ref*_) and *ΔS(T*_*ref*_) are estimated from the simulation at 350 K, and *ΔC*_*p*_ = 1.98±0.86 kJ/molK is estimated following refs 72, 73.

### Protein expression and purification

The Aβ40 peptide (MDAEFRHDSGY EVHHQKLVFF AEDVGSNKGA IIGLMVGGVV) was expressed in the *E. coli* BL21 Gold (DE3) strain (Stratagen) and purified as described previously with slight modifications^74^. Briefly, the purification procedure involved sonication of *E. coli* cells, dissolution of inclusion bodies in 8 M urea, ion exchange in batch mode on diethylaminoethyl cellulose resin. Eluates were analyzed using SDS-PAGE for the presence of the desired protein product. The fractions containing the recombinant protein were combined and then frozen using liquid nitrogen and lyophilized. Solutions of monomeric Aβ40 were prepared by dissolving lyophilized peptide in 6 M GuHCl. Monomeric Aβ40 was purified from potential oligomeric species and salt using a Superdex 75 10/300 GL column (GE, Healthcare), and was eluted in 20 mM sodium phosphate buffer, pH 8. The peptide concentration was determined by absorbance at 275 nm using ε_275_ = 1400 m^−1^ cm^−1^.

### Size exclusion chromatography and hydrodynamic analyses

The apparent molecular mass (*M*_*app*_) of proteins eluted from gel filtration columns was deduced from a calibration carried out with LMW calibration kits (GE, Healthcare). The value of *R*_*H*_ of a protein can be deduced from *M*_*app*_ (as seen by gel filtration)^52^.

The values of *R*_*H*_ (in Å) of the folded and unfolded states in urea, and in a pre-molten globule (PMG) with a molecular mass (*M*) (in Daltons) can be estimated as^53^


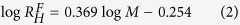



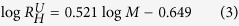



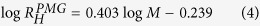


We also used empirical equations derived from NMR diffusion experiments^54^ to estimate *R*_*H*_ of folded and unfolded proteins based on Aβ40 number of amino acids


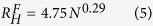



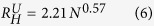


The compaction index (CI) was calculated according to^55^


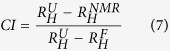


### Pulse-field gradient NMR diffusion experiments

The value of *R*_*H*_ of Aβ40 was accurately determined using pulsed-field gradient NMR experiments^54^. NMR spectra were recorded on a Bruker Avance 700 MHz Ultrashield spectrometer equipped with a TXI cryoprobe. The spectra were recorded in the temperature range 5–40 °C with data pitch of 5 °C. Tube of 3 mm were used in order to minimise internal convection. We used both 2D Stimulated Echo experiment using bipolar gradients and WATERGATE pulse for water suppression (stebpgp1s19 pulse sequence) and 2D sequence for diffusion measurement using double stimulated echo for convection compensation and LED using bipolar gradient pulses for diffusion using 3 spoil gradients (dstebpgp3s pulse sequence^75^,^76^ and these two measurements leads to the same results. The gradient was varied between 5% and 95% and a total number of 16 scans were acquired. The gradient time (δ) and the diffusion time (∆) were set so that the signal intensity varied from 95% to 5% as the gradient strength increases. Data processing was performed using NMRPipe^77^.

Briefly, in these experiments, the intensity of the observed signal decreases with the strength of the gradient applied as


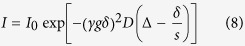


with γ the gyromagnetic ratio of ^1^H, *g* the gradient strength, and *D* the diffusion coefficient. The value of *R*_*H*_ was deduced from the diffusion coefficient according to the Stokes-Einstein equation


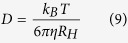


where *k*_*B*_ is the Boltzmann constant, *T* is the temperature in Kelvin and *η* is the viscosity of the solution. Since the absolute value of *D* depends on the temperature and viscosity of the solution (Eq. 9), we used 4,4-dimethyl-4-silapentane-1-sulfonic acid (DSS) or dioxane as internal radius standard^54^,^78^ and references therein cited). Accordingly, the value of *R*_*H*_ of Aβ40 was compared to the one of DSS or dioxane (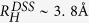, 
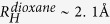
) as^79^


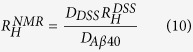


with *D*_*DSS*_ and *D*_*Aβ*40_ being the diffusion coefficients of DSS and of the Aβ40 peptide, respectively. The hydrodynamic radius of DSS was calibrated by performing the diffusion measurements in the presence of hen lysozyme whose hydrodynamic radius was set to 15.3 Å, as determined by SAXS^80^. The ratio between *D*_*DSS*_ and *D*_*Aβ*40_ was calculated for each temperature by fitting the attenuation curves of the signal of both Aβ40 and DSS using equation (8) (Fig. S1).

### Circular dichroism

CD experiments were carried out using a Jasco J-810 spectropolarimeter equipped with a Peltier holder in 20 mM sodium phosphate pH 8.0. CD spectra were measured at protein concentration of 20 μM, with a scanning speed of 20 nm/min and a data pitch of 0.2 nm. Spectra were averaged from three scans and smoothed using the “means-movement” smoothing procedure implemented in the Spectra Manager package. The contribution of buffer was subtracted from experimental spectra. Mean ellipticity values per residue (MRE) were calculated as


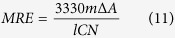


where l is the path length (0.1 cm), m the molecular mass (4,461 Da) and *C* the protein concentration expressed in mg/mL.

Heat-induced structural transitions were monitored between 5 and 95 °C at a rate of 5 °C/min. CD spectra were acquired every 5 °C with a response time of 2 s and a bandwidth of 1 nm. The experimental data in the 190–250 nm range were analyzed using the CDSSTR deconvolution method that is implemented in the CDPro software package with the reference protein set 7 (http://lamar.colostate.edu/~sreeram/CDPro/main.html)^56^.

### Thermal denaturation/renaturation using Tyrosine fluorescence spectroscopy

Fluorescence spectra of the single tyrosine were carried out using a Cary Eclipse (Varian) equipped with a Peltier unit for temperature control. All measurements were obtained at 100 μM in 20 mM sodium phosphate pH 8.0 using a 1-cm quartz cuvette. The excitation wavelength was 275 nm, and the emission spectra were recorded between 290 and 350 nm, with 5 nm excitation and 10 nm emission bandwidths. Experimental fluorescence intensities were corrected by subtracting the spectrum obtained with the buffer. Heat-induced experiments were monitored between 10 and 100 °C every 10 °C after a delay of 5 min at each temperature. Data were analyzed by plotting either the full spectra or the relative fluorescence intensities at the maximum of emission as a function of temperature.

### Database of metastable partially folded conformations of the Aβ40 peptide

From the free energy landscape that we determined we have extracted a set of partially folded metastable structures (available upon request). These structures could for example be used for deriving coarse grained models to study protein aggregation, or as targets to design inhibitors capable of binding the Aβ40 monomer to prevent its aggregation in drug discovery.

## Additional Information

**How to cite this article**: Granata, D. *et al.* The inverted free energy landscape of an intrinsically disordered peptide by simulations and experiments. *Sci. Rep.* **5**, 15449; doi: 10.1038/srep15449 (2015).

## Supplementary Material

Supplementary Information

## Figures and Tables

**Table 1:** Hydrodynamic radius (*R*_*h*_) of Aβ40 obtained from SEC and NMR diffusion experiments at different temperatures, as well as the predicted values for various conformational states (F: natively folded^54^, U: urea unfolded^53^, PMG: pre-molten globule^53^).

	SEC 20 °C	NMR 5 °C	NMR 25 °C	NMR 40 °C	F	U	PMG
*R*_*H*_ (Å)	16	16.9±0.8	15.9±1.0	15.5±1.8	12.4–13.9	18.0–18.4	17
CI		0.21	0.38	0.45			

We also report the compaction index (CI) values, which were calculated using reference *R*_*h*_ values for unfolded and natively folded proteins^53^. For comparison, the average *R*_*h*_ value of the simulated ensemble is 17.8 Å, as derived from the average *R*_*g*_ value of 12.3 Å (Fig. 2) using a fitting procedure^18^.
