# Supplementary material for: The inverted free energy landscape of an intrinsically disordered peptide by simulations and experiments
Source: Sci Rep. 2015 Oct 26;5:15449. doi: 10.1038/srep15449 (PMC4620491; doi:10.1038/srep15449)
Supplement: Supplementary Information [file srep15449-s1.pdf]

SUPPORTING INFORMATION

The inverted free energy landscape of an intrinsically disordered peptide by simulations and experiments

Daniele Granata[1,2], Fahimeh Baftizadeh[3], Johnny Habchi[4], Celine Galvagnion[4], Alfonso De Simone[5], Carlo Camilloni[4], Alessandro Laio[1] and Michele Vendruscolo[4,*]

[1]*International School for Advanced Studies (SISSA), 34136 Trieste, Italy*

[2]*Institute of Computational and Molecular Science (ICMS), Temple University, Philadelphia, PA 19122, USA*

[3]*Department of Chemical Engineering, Massachusetts Institute of Technology (MIT), Cambridge, MA 02139, USA*

[4]*Department of Chemistry, University of Cambridge, Lensfield Road, Cambridge CB2 1EW, UK*

[5]*Division of Molecular Biosciences, Imperial College London, London SW7 2AZ, UK*

[*]Corresponding author: mv245@cam.ac.uk

Atom type	Ensemble-averaged chemical shifts	Random coil chemical shifts	Prediction error
HA	0.10	0.08	0.25
HN	0.30	0.28	0.46
N	1.25	1.45	2.36
CA	0.35	0.36	0.88
CB	0.45	0.28	0.97

Table S1. Average deviations between the experimental chemical shifts of the Aβ40 peptide (at 5 °C and 10 mM ionic strength[1]) and the average chemical shifts back-calculated by SPARTA+[2] from the ensemble generated in this work using molecular dynamics simulations (at 77 °C and 0 mM ionic strength). In the second column the same comparison is performed using random coil chemical shifts[3]. The typical error of the predictor is also reported. All the numerical values are expressed in ppm.

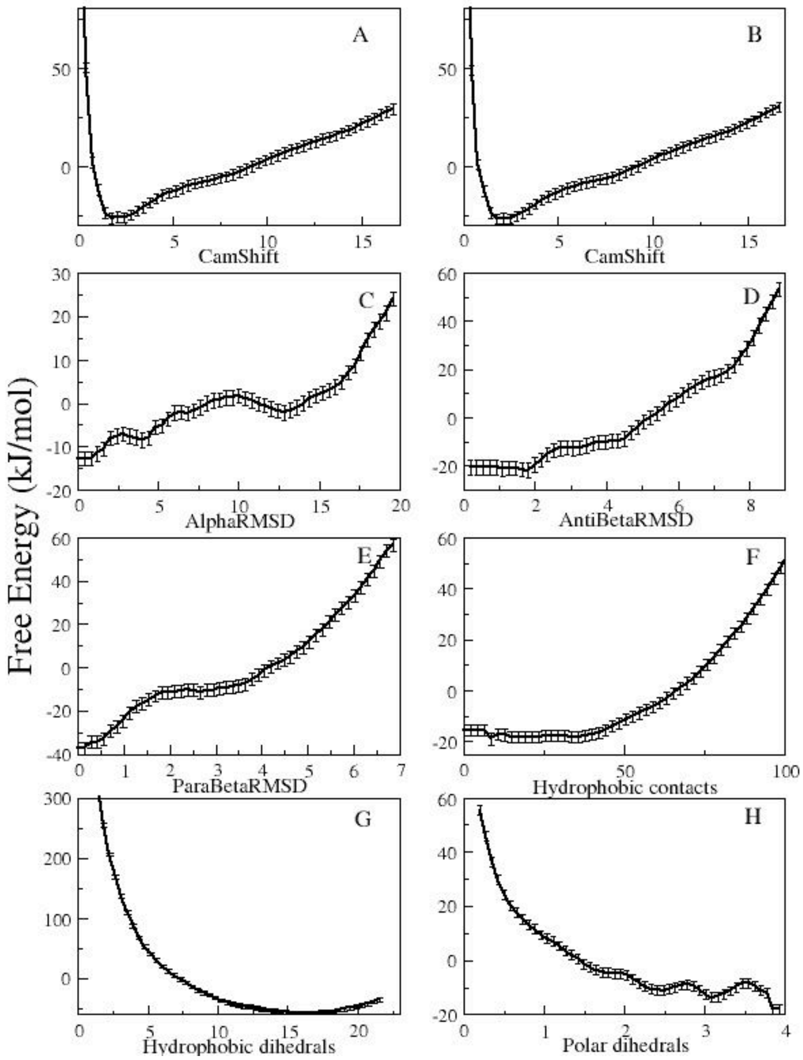

Figure S1. One-dimensional profiles for the bias potentials acting on the eight replicas used in the simulations. Each replica is associated with a specific collective variable: (A,B). The CamShift collective variable, which measures the deviations between experimental and calculated chemical shifts. Notice that in the setup we use two replicas are biased by this variable; (C) AlphaRMSD; (D) AntiBetaRMSD; (E) and ParaBetaRMSD (E) for the corresponding secondary structure content; (F) Hydrophobic contacts, which counts the number of contacts between the heavy atoms of the hydrophobic residues and measures the degree of compactness of the protein; (G,H), deviation from the average χ_1 and χ_2 torsion angles values for the hydrophobic and polar residues side chains. All the free energy profiles are shown with the relative error bars estimated by block analysis, which are in all cases below 2.9 kJ/mol, demonstrating the high convergence reached by the metadynamics simulation.

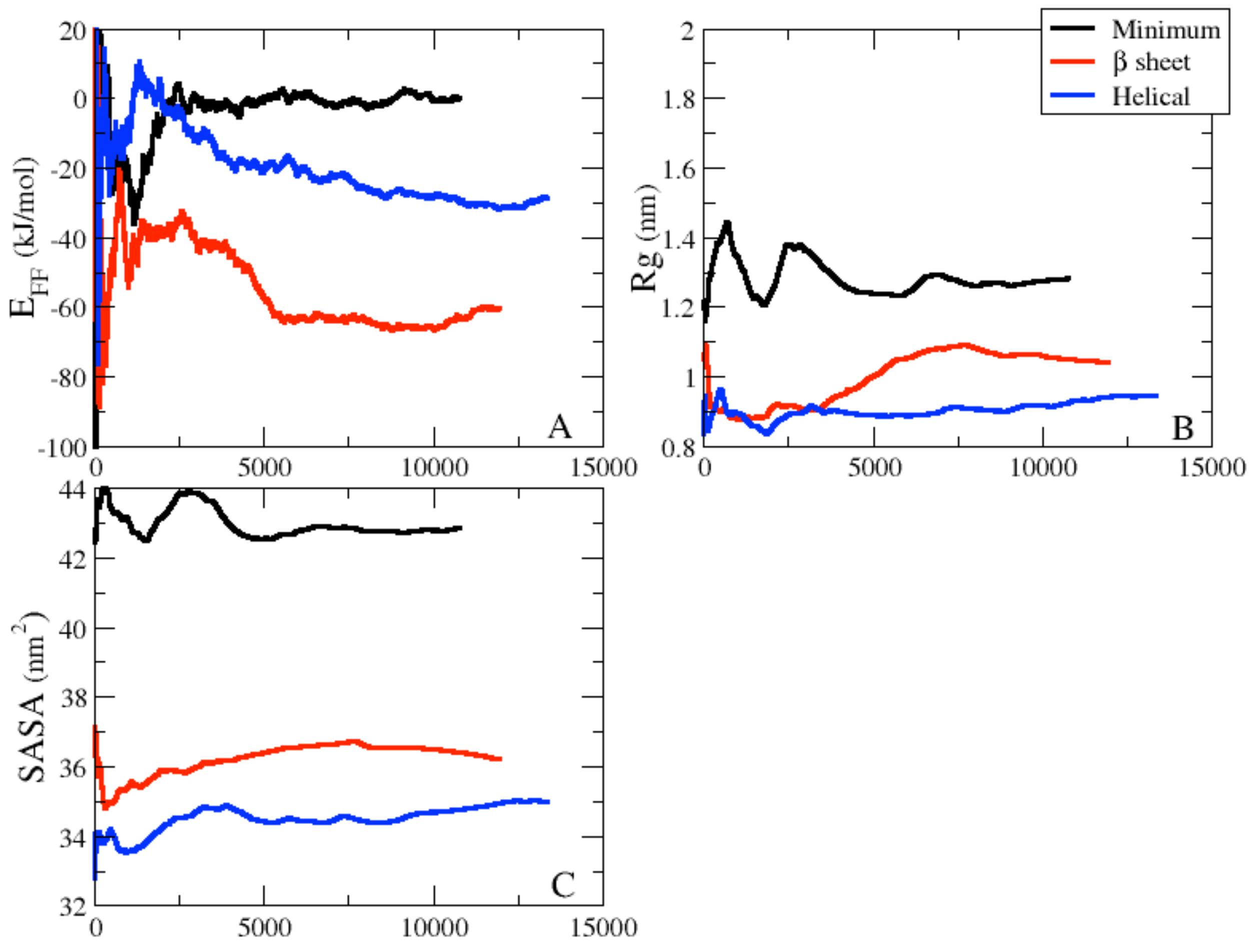

Figure S2: Convergence of the enthalpy (total force field energy, E_{FF}), radius of gyration (Rg) and solvent accessible surface area (SASA) from the three simulation runs starting from different conformations: the global disordered minimum (black line), β-strand (red line) and helical one (blue line). The corresponding structures are highlighted with dots of the same color in **Fig. 2** in the main text. See Methods in the main text for details about the simulation.

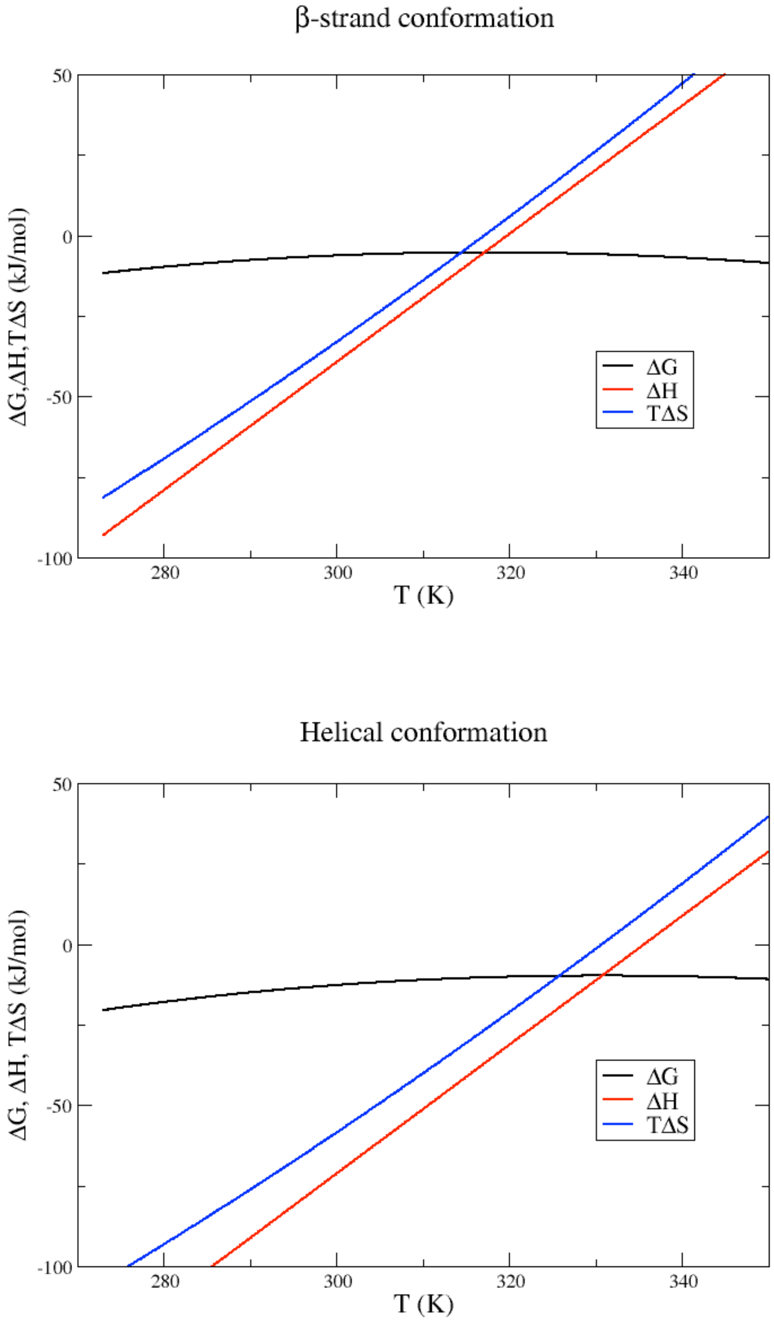

Figure S3: Temperature dependence of the free energy (black line), enthalpy (red) and entropy (blue) differences between the global disordered minimum and respectively β-strand and α-helical conformation one (see Methods in the main text for details about the estimation).

Supplementary References

1. Hou, L. M. *et al.* Solution NMR studies of the Abeta(1-40) and Abeta(1-42) peptides establish that the Met35 oxidation state affects the mechanism of amyloid formation. *J. Am. Chem. Soc.* **126**, 1992-2005, (2004).
2. Shen, Y. & Bax, A. Sparta+: A modest improvement in empirical NMR chemical shift prediction by means of an artificial neural network. *J. Biomol. NMR* **48**, 13-22, (2010).
3. De Simone, A., Cavalli, A., Hsu, S.-T. D., Vranken, W. & Vendruscolo, M. Accurate random coil chemical shifts from an analysis of loop regions in native states of proteins. *J. Am. Chem. Soc.* **131**, 16332-16333, (2009).
